# A Simple, Safe, and Effective Method for Preparing Autologous Bio-Based Fibrin Glue for Ophthalmic Use

**DOI:** 10.3390/pharmaceutics14112325

**Published:** 2022-10-28

**Authors:** Luis Fernández-Vega-Cueto, Mairobi Persinal-Medina, Natalia Vázquez, Manuel Chacón, Belén Alfonso-Bartolozzi, Sergio Alonso-Alonso, Teresa Sánchez, Silvia Berisa-Prado, Laura María Martínez-López, Jesús Merayo-Lloves, Álvaro Meana

**Affiliations:** 1 Instituto Oftalmológico Fernández-Vega, Avenida Doctores Fernández-Vega, 33012 Oviedo, Spain; 2 Instituto de Investigación Sanitaria del Principado de Asturias (ISPA), Avenida del Hospital Universitario, 33011 Oviedo, Spain; 3 Instituto Universitario Fernández-Vega, Fundación de Investigación Oftalmológica, Universidad de Oviedo, Avenida Doctores Fernández Vega, 33012 Oviedo, Spain; 4 Unidad de Bioterio e Imagen Preclínica, Universidad de Oviedo, Avenida Julián Clavería, 33006 Oviedo, Spain; 5 Unidad de Ingeniería Tisular, Centro Comunitario Sangre y Tejidos de Asturias (CCST), Unidad 714 CIBERER, Calle Emilio Rodríguez Vigil, 33006 Oviedo, Spain

**Keywords:** fibrin glue, tissue glue, sealant, tissue adhesive, ophthalmology

## Abstract

This study proposes a method to prepare autologous bio-based fibrin glue (FG) for use in ophthalmic surgery. FGs containing three fibrinogen concentrations and a thrombin concentrate were prepared using human blood from five donors (FG1: physiological fibrinogen concentration; FG2 and FG3: concentrated fibrinogen). The adhesion strength was tested, and the clinical safety and efficacy were studied in rabbit eyes in conjunctival surgery. A commercial FG was used as a control. From each donor, 2 mL of FG was prepared, containing 1 mL of 3.49 ± 0.78 (FG1), 17.74 ± 4.66 (FG2), or 47.46 ± 9.36 mg/mL (FG3) of fibrinogen and 1 mL of 2248.12 ± 604.20 UI/mL of thrombin. The average adhesion strength increased with the fibrinogen concentration, from 1.49 ± 0.39 kPa (FG1) to 3.14 ± 1.09 kPa (FG3). FG1 showed poor results when used for autograft adhesion. In contrast, the conjunctival autografts were successfully grafted using FG2 and FG3, revealing equivalent adhesion properties compared with commercial FG, but with less inflammation. In conclusion, FGs could be prepared on demand within minutes from small volumes of human blood, using a method that results in FGs which exhibit good adhesion capacity and are also safe and effective in a preclinical study.

## 1. Introduction

Sutures are medical devices used to sew body tissues together to close an injury or a surgical incision. There are many suturing techniques and suture materials available; surgeons are responsible for selecting the appropriate choice in each case during clinical interventions. An ideal suture is one which is easily manageable, non-allergenic, and affordable, and does not promote post-surgical pain, inflammation, and infection [1].

In recent years, tissue adhesives (also known as tissue glues or sealants) have gained popularity in surgical procedures in order to replace sutures in certain situations, due to their ease of use, minimal pain to the patient, lack of tissue penetration, and rapid application in comparison to sutures [1,2,3]. For ophthalmic use, tissue adhesives are classified in two basic categories [1,4]: synthetic (commonly based on n-butyl-2-cyanoacrylates [5] and polyethylene glycol [6,7]) and biological (commonly based on fibrin [5,7,8]).

Synthetic adhesives are used due to their high adhesion strength, instant adhesion, and no risk of transmitting infectious agents. However, synthetic adhesives show higher cytotoxicity than biological adhesives, and when used often result in abnormal scarring because they are not easily degraded during the healing process [6]. On the other hand, biological adhesives are progressively degraded by cells without toxicity and are well tolerated in the eye [4,9,10]. In addition, biological adhesives show numerous advantageous properties compared with synthetic adhesives. They allow enough working time before polymerizing, are able to reach adequate adhesion strength to maintain wound integrity, induce less inflammation, and are clear enough to allow vision [1]. However, biological adhesives also present several downsides compared with synthetic adhesives, because biological adhesives may carry the risk of transferring infectious agents and show limited adhesion strengths [11]. Nevertheless, because in ophthalmology the demand of adhesion strength is low, biological adhesives represent a more interesting clinical tool than synthetic adhesives. 

In recent years, bio-based fibrin glue (FG) adhesives have increasingly been used in different ocular procedures such as pterygium surgery, corneal flap tears and dislocations, treatment of corneal perforations and melts, or cataract and retinal surgeries [5,12,13]. These FGs include a fibrinogen component and a thrombin component, both prepared by processing plasma, which polymerize by mimicking the final steps of fibrin coagulation cascade [1]. Most of the FGs used in clinical practice are commercially available [9]; however, each one slightly differs in its composition (concentrations and excipients) and mechanisms of delivery. All commercially available FGs are made from human pooled plasma of different blood donors and contain high concentrations of fibrinogen (40–110 mg/mL [14]) which consequently produce firm clots [2]. However, the allogeneic origins and the high cost of these commercial adhesives represent the main limitations for their wide acceptance.

As alternatives to the use of expensive allogenic FGs, non-commercial autologous FGs can be prepared by combining citrated blood plasma with calcium chloride [10,15,16]. However, physiological concentrations of fibrinogen and thrombin are variable in the population, and as result, autologous FGs have low adhesion strength, produce less firm clots, and the coagulation time is higher than in commercial FGs [10,15,16,17].

To increase adhesion strength, numerous techniques [18,19,20,21,22,23] have been used to prepare FG, either from allogeneic or autologous plasma. Fibrinogen can be concentrated from plasma by cryoprecipitation or chemical precipitation. Thrombin can be purified from plasma by chromatography or acid precipitation. Additionally, autologous serum can be used as the thrombin component.

In this study, we have developed a new method to prepare autologous FGs based on concentrated fibrinogen and thrombin using small amounts of human blood obtained by venipuncture. We describe the production method of FGs, the characterization of their mechanical properties, and the preclinical results after applications as conjunctival adhesives for autograft surgery in an animal model. Overall, we propose that, with the method described here, FGs could be manufactured in minutes in a hospital pharmacy prior to use in surgical procedures, representing a simple, safe, and effective alternative to commercial FGs.

## 2. Materials and Methods

### 2.1. Blood Collection

Whole human blood was collected from healthy volunteers (*n* = 5; age range 23–47 years) by venipuncture after obtaining written informed consent. From each donor, 140 mL of blood was collected into 9 mL tubes containing 3.8% (*w*/*v*) sodium citrate (Vacuette^®^, Greiner Bio-One, Madrid, Spain). Blood tubes were centrifuged at 580× *g* for 8 min at room temperature to separate the plasma.

### 2.2. Preparation of FGs

FGs were prepared at three different fibrinogen concentrations (FG1, FG2, and FG3), maintaining similar thrombin concentrations.

Isolation of thrombin. The thrombin solutions were prepared according to the method described by Quarmby J et al. [23], with some modifications. Briefly, 10 mL of plasma was diluted in 0.04% glacial acetic acid (1:4) (Guinama, Valencia, Spain) at room temperature, and the solution was centrifuged at 1500× *g* for 20 min at 22 °C. The pellet was resuspended in 1 mL pH 6.5 buffer containing 0.9% sodium chloride, 0.03% sodium bicarbonate (Fagron Iberica SAU, Barcelona, Spain), and 25 mM calcium chloride (B. Braun Medical S.A., Barcelona, Spain). The mixture was vortex-mixed for 2 min, incubated at room temperature for 15 min to precipitate the fibrin, and filtered through a 40 µm nylon cell strainer (Thermo Fisher Scientific, Waltham, MA, USA). The precipitated fibrin retained in the filter was removed, and the thrombin concentrate was collected and loaded into a 2 mL syringe (BD Biosciences, San Jose, CA, USA). 

Isolation of fibrinogen. To prepare FG1, 1 mL of plasma was directly loaded into a 2 mL syringe. To prepare FG2 and FG3, the fibrinogen was concentrated by the addition of protamine sulphate (Sigma-Aldrich, St. Louis, MO, USA), as described by Steven M. et al. [17]. Briefly, 10 mL of protamine sulphate (40 mg/mL) was added to 30 mL of plasma (final concentration 10 mg/mL) at room temperature, mixed, and centrifuged at 1000× *g* for 5 min at 22 °C. After centrifugation, the supernatant was discarded, and the precipitate was dissolved in 3 mL (FG2) or 1 mL (FG3) of 0.2 M sodium citrate (37 °C, pH 7.4) (Guinama), and loaded into 2 mL syringes.

Fibrinogen and thrombin syringes were loaded in a dual-barrel 2 mL cartridge (ratio 1:1) with a mixing tip. As a control group for subsequent analysis, a commercially available FG was used as a reference (TISSEEL; Baxter, IL, USA).

### 2.3. Fibrinogen Quantification

Fibrinogen concentrations were measured in each FG group (*n* = 5) using the Clauss method [24] with an STA-Compact-Max^2^ analyzer (Stago, Asnières sur Seine, France). All assays were performed according to the manufacturer’s instructions, and the tests were run in duplicates.

### 2.4. Thrombin Activity Quantification

The thrombin activities were determined in each FG group (*n* = 5) on a Behring Fibrintimer II (Dade Behring, Deerfield, IL, USA). Firstly, a standard curve was constructed for known 100 µL concentrations of human fibrinogen (2.11 mg/mL) with 200 µL of commercial thrombin (Siemens AG, Munich, Germany) at different activities, from 50 to 100 UI/mL, at 37 °C. Then, the activity of thrombin’s samples was determined in duplicate by adding 200 µL of diluted (1:5 to 1:40) thrombin to 100 µL of this fibrinogen. Finally, the thrombin activities were interpolated using the standard curve.

### 2.5. Adhesion Strength Measurement

**Figure 1:**
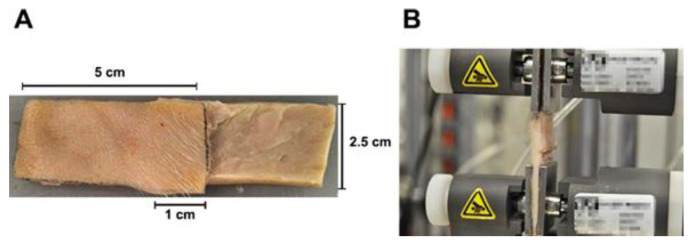
Adhesion strength assay. (**A**) Samples of porcine skins glued with FGs or commercial FG, indicating its dimensions (5 cm length, 2.5 cm width, and 1 cm overlap) according to the ASTM manual F2255–05 [25]. (**B**) Adhesion strength test. ASTM: American Society for Testing and Materials; FG: bio-based fibrin glue.

The adhesion strengths of FGs (*n* = 5 for each group) and commercial FG were assessed in duplicate using the standard test method for strength properties of tissue adhesives in lap-shear by tension loading, following the methodology described by the American Society for Testing and Materials (ASTM) in designation F2255–05 [25]. Briefly, porcine skin was cut into strips, each approximately 5 cm long and 2.5 cm wide. FGs were applied (0.4 mL) to an area of 2.5 cm^2^, as shown in Figure 1. The overlapping joint was formed and allowed to cure while being held in place with a 5 N weight for 15 min at 22 °C. Strips were inserted in an INSTRON 5548 Universal Testing System (Instron, Norwood, MA, USA) and pulled at a rate of 5 mm/min until the joint failed or tissue rupture occurred.

### 2.6. Preclinical Study

#### 2.6.1. Animals

New Zealand white rabbits (males, 12 weeks, 3.5–4.0 kg) were purchased from Granja San Bernardo (Navarra, Spain). All the animals were treated in accordance with The Association for Research in Vision and Ophthalmology (ARVO) Resolution on the Use of Animals in Ophthalmic and Vision Research. The protocols were approved by the Committee on the Ethics of Animal Experiments of the University of Oviedo and the Animal Production and Health Service of Asturias (Spanish registration code PROAE 36/2019).

#### 2.6.2. Surgical Procedure

FGs were tested as conjunctival adhesives for autograft surgery, involving the transference of a free graft of bulbar conjunctiva to cover the exposed scleral tissue [26]. The efficacy of the different FGs was evaluated in 4 animals per group.

Anesthesia:

All surgeries were performed under general anesthesia. Anesthesia was induced by the intravenous administration of 0.2 mg/kg medetomidine (Domtor^®^, Orion Corporation, Espoo, Finland) and 1–2 mg/kg diazepam (Valium^®^ 10, Hoffmann-La Roche, Basilea, Switzerland); rabbits were maintained under 3% isoflurane inhalation. Additionally, double anesthetic colircusi^®^ (0.1% tetracaine and 0.4% oxybuprocaine) was applied 10 min before the intervention (Colirusi^®^, Alcon Healthcare SA, Barcelona, Spain).

Receptor zone preparation:

The bare scleral bed was exposed by dissection of the temporal conjunctiva and the underlying Tenon’s capsule, as described subsequently. A vasoconstrictive solution (1 mg/mL adrenaline, B. Braun Medical SA) was injected to balloon out the conjunctiva and Tenon’s capsule, then excised with Wescott scissors, exposing a 4 × 5 mm bare scleral bed. 

Graft dissection from donor zone:

A 5 × 6 mm free autograft was taken from the superior bulbar conjunctiva, marking the epithelial side and the juxtalimbar border to prevent graft inversion and disorientation. Vasoconstrictive solution (0.1 mL) was injected under the donor conjunctiva to balloon out the area of the autograft and separate it from the underlying Tenon’s capsule. Subsequently, the conjunctiva was carefully dissected away from the Tenon’s capsule, taking special care to prevent buttonholes and graft rollover. 

Grafting using FGs and postsurgical care:

The graft was placed on top of the cornea, stretched, oriented, and then adhered on the bare scleral bed with 0.4 mL of FG as follows. A 0.2 mL volume of FG was applied on the bare sclera and spread out using a cannula, and the graft was immediately transferred onto the bare sclera. An additional 0.2 mL of FG was applied to the edges of the graft limiting with the healthy conjunctiva and the cornea. After a drying period of 5 min, passive blinking was induced to verify graft attachment. Finally, tarsorrhaphy was performed with 4-0 nylon suture to prevent ocular damages attributable to the animal behavior. Sutures were removed after 3 days and non-attached clots of FG were removed with eye spears (Weck-Cel^®^, Medical Mix SLU) and balanced sterile saline washes (BSS^®^, Medical Mix SLU, Barcelona, Spain).

Postoperative treatment consisted of daily subcutaneous administration of antibiotic (5–10 mg/kg enrofloxacin; ALSIR^®^, Ecuphar, Barcelona, Spain) and analgesia (0.3 mg/kg meloxicam; Metacam^®^, Boehringer Ingelheim, Barcelona, Spain and 0.01–0.05 mg/kg buprenorphine; Bupaq^®^, Richter Pharma AG, Wels, Austria) for 7 days after surgery. Additionally, topical eye drops of antibiotic (0.3% tobramycin; Tobrex^®^ Alcon Healthcare SA) and glucocorticoid (0.1% dexamethasone; Maxidex^®^, Alcon Healthcare SA) were administered thrice-daily from day 3 to day 14.

#### 2.6.3. Clinical Follow-Up

All the rabbits were examined from the tarsorrhaphy opening until day 14. Clinical evaluation consisted of the evaluation of presence of FG residues, dehiscence, inflammation, hemorrhage, retraction, integration, and vascularization. At the end of follow-up, all rabbits were euthanized by sedation with medetomidine and diazepam (as described above), followed by an intravenous overdose of sodium pentobarbital. Finally, eyes were enucleated and fixed in 4% paraformaldehyde for 24 h for histological evaluation.

### 2.7. Histology

The grafted area was dissected from enucleated eyes after fixing. The samples were embedded in paraffin, and tissue sections (5 µm) were prepared. Deparaffinized tissue sections were stained with hematoxylin–eosin (H&E), dehydrated, cleared, and mounted for examination under a Leica DM6000B bright-field microscope fitted with a DFC310FX camera (Leica, Wetzlar, Germany). Moreover, an immunofluorescence against interleukin-6 (IL-6; Ref: ab6672, Abcam, Cambridge, UK) was performed. Briefly, deparaffinized tissue sections were rinsed with phosphate-buffered saline (PBS) solution twice for 10 min and permeabilized in a PBS solution containing 0.3% Triton X-100 for another 5 min. Next, the samples were incubated at 4 °C overnight with primary antibody (1:100) containing 10% normal goat serum (Life Technologies, Carlsbad, CA, USA) as a blocking agent. Immunolabeled cells were visualized by indirect immunocytochemistry and stained with 4′, 6-DiAmidino-2-PhenylIndole (DAPI) to visualize nuclei. Samples were examined using a Leica DM6000B fluorescence microscope (Leica, Wetzlar, Germany). Tissue morphology, inflammatory reaction, vascularization, the presence of eosinophilic FG residues, granulation tissue, and scar formation were evaluated in each sample.

### 2.8. Data Analysis

Graphic representations and statistical evaluations were performed using IBM SPSS Statistics version 22 (IBM Corp, New York, NY, USA). The normal distribution of tested values was assessed by the Shapiro–Wilk method. Significant differences among defined groups were assessed using the Mann–Whitney U test. Significant differences were considered when *p* < 0.05. The quantitative data are expressed as the mean ± SD.

## 3. Results

### 3.1. Characteristics of FGs

Thrombin and fibrinogen were successfully isolated and concentrated from single-donor blood samples following the methods described above.

**Figure 2:**
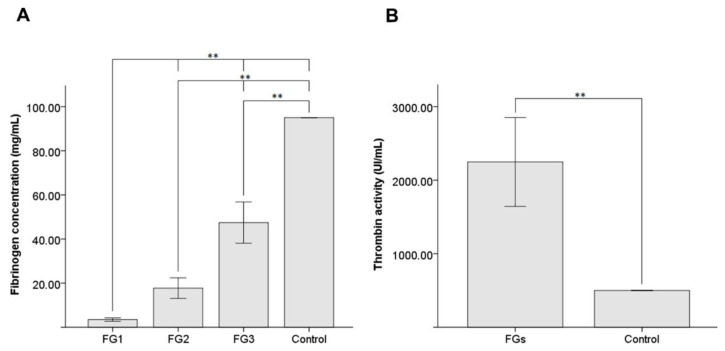
Mean fibrinogen concentration (**A**) and thrombin activity (**B**) obtained in FG1, FG2, and FG3, and a statistical comparison with the commercial FG as a control. **: *p*-value < 0.01. Data are shown as the mean ± SD. FG: bio-based fibrin glue.

Mean fibrinogen concentration values are shown in Figure 2A for each group. The mean fibrinogen concentration was 3.49 ± 0.78 mg/mL in FG1, 17.74 ± 4.66 mg/mL in FG2, and 47.46 ± 9.36 mg/mL in FG3. Statistically significant differences were shown for fibrinogen concentrations between FG1, FG2, and FG3 (*p*-values = 0.009). Moreover, statistically significant differences (*p*-values = 0.005) were shown between our FGs and the commercial FG (91 mg/mL) used as a control.

Thrombin was isolated, activated, and concentrated up to 2248.12 ± 604.20 IU/mL mean enzymatic activity (Figure 2B). Statistically significant differences (*p*-value = 0.005) were shown with the commercial FG (500.00 ± 0.00 IU/mL) used as a control. 

**Figure 3:**
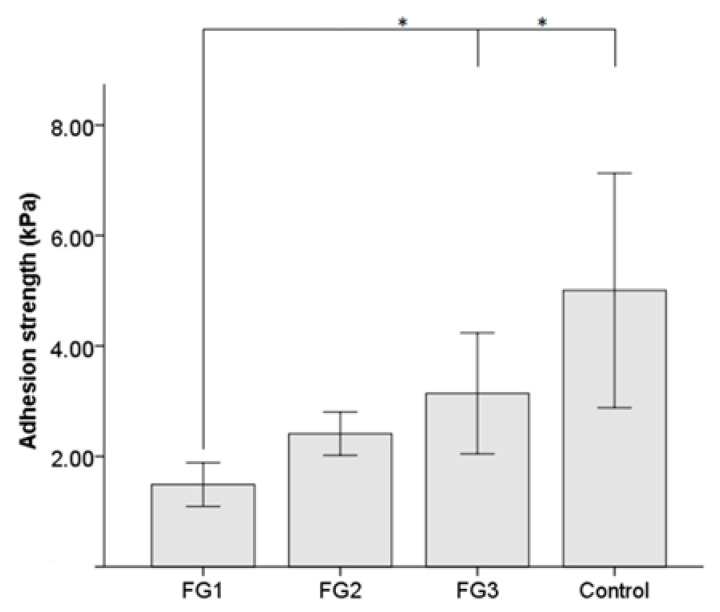
Mean adhesion strength obtained in FG1, FG2, and FG3 and a statistical comparison with the commercial FG as a control. *: *p*-value < 0.05. Data are shown as the mean ± SD. FG: bio-based fibrin glue.

Mean adhesion strength values are shown in Figure 3 for each group. Maximum adhesion strengths were achieved using the commercial FG (5.01 ± 2.12 kPa) used as a control, followed by FG3 (3.14 ± 1.09 kPa) and FG2 (2.41 ± 0.39 kPa), representing 62.8% and 48.2% of the mean adhesion strength of the commercial FG, respectively. FG1 exhibited statistically significantly lower adhesion strength (1.49 ± 0.39 kPa) than FG3 (*p*-value = 0.025) and the control group (*p*-value = 0.018). No other significant differences were found between the groups.

### 3.2. Preclinical Study

#### 3.2.1. Intraoperative and Clinical Observations

**Figure 4:**
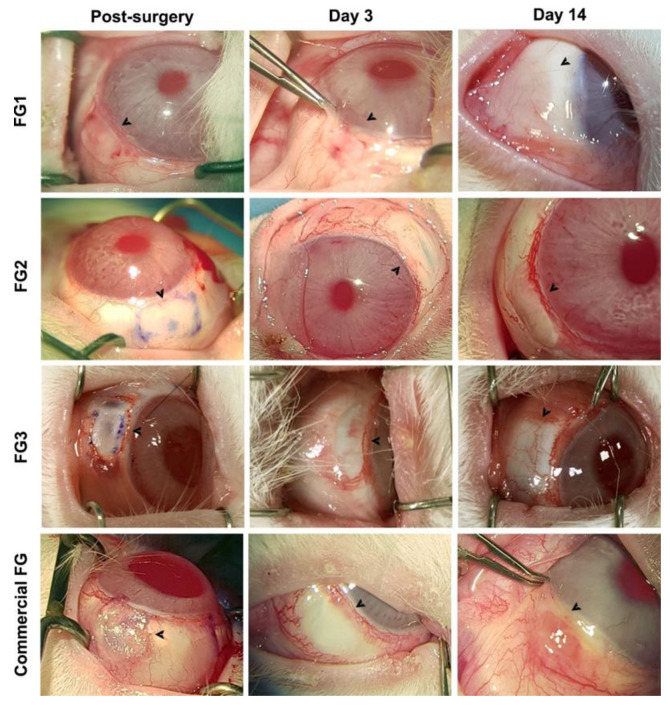
Clinical evolution of bulbar conjunctiva autografts on a bare scleral bed transplanted using different FGs and the commercial FG. Arrows show the grafted area in the bulbar conjunctiva. FG: bio-based fibrin glue.

Intraoperative and clinical observations showed that FG1, FG2, and FG3 allowed the application of 0.2 mL at different intervals with high extrusion control and placement of clots (Figure 4). FGs instantly formed a firm and sticky clot, but allowed enough working time before inducing firm adhesion. Autografts glued with FG2 and FG3 were successfully attached and exhibited adequate adhesion strength to maintain wound integrity without folds and dehiscences during blinking. However, autografts glued with FG1 slightly wrinkled during blinking and showed dehiscences in the posterior edge. On the other hand, commercial FG also induced firm adhesion; however, the resultant working time was insufficient and irregular clots were formed.

After 3 days, none of the groups showed graft dehiscence from the scleral bed. Subconjunctival hemorrhage around the graft was observed in some cases in every group, but it resolved spontaneously in 3–7 days without complications.

At the end of follow-up, all the autografts appeared vascularized and integrated within the surrounding conjunctival tissue without adverse effects on the cornea or conjunctiva. There was no significate inflammation in the FG1, FG2, and FG3 groups, whereas commercial FG showed mild–moderate sings of inflammation in the graft and the surrounding conjunctiva.

#### 3.2.2. Histological Evaluation

**Figure 5:**
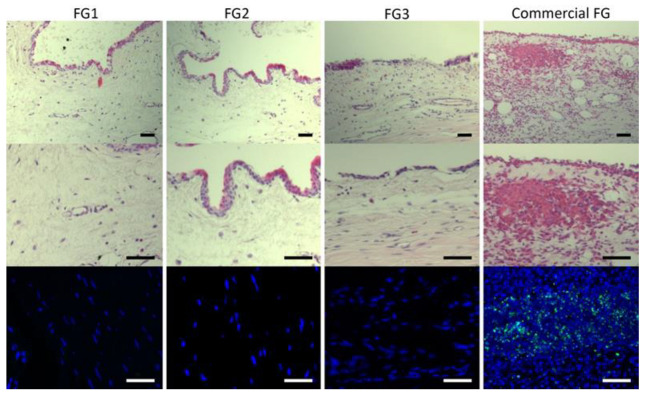
H&E and IL-6 immunofluorescence stains of a representative sample of each FG group. No inflammatory cells and no edema were observed in FG1, FG2, or FG3 samples. Commercial FG showed inflammatory cells and edema. Scale bars: 50 µm. FG: bio-based fibrin glue; IL-6: interleukin-6.

The autografts showed an adequate integration into the bulbar conjunctiva, where a continuous layer of stratified squamous non-keratinized epithelium was observed in all groups with successful integration of the glued autograft into the bulbar conjunctiva (Figure 5). However, several differences were observed at the stromal level in the grafted area between groups. Immune infiltrates were absent or minimally present in FG1, FG2, and FG3 groups, whereas commercial FG group showed mild to severe immune reactions and stromal edema. These results were confirmed by IL-6 immunofluorescence (Figure 5).

## 4. Discussion

The use of FGs over sutures have shown numerous advantages in ophthalmic surgeries [1], such as in amniotic membrane fixation [27,28,29], lamellar keratoplasty [9,30], Descemet membrane perforation [31], strabismus surgery [32], Faden operation [33], or conjunctival autograft adhesion in the surgical treatment of pterygium [34,35].

Several companies are already commercializing FGs manufactured under rigorous quality control procedures, designed for a wide range of therapeutic indications [1]. However, there is not an FG specifically designed for use in ophthalmology, where the demands for adhesion strength and volume/surface ratio are low; the disadvantages of the use of commercial FGs in this field have already been highlighted [36].

Autologous FGs can easily be produced in the operative field [37,38] using physiological concentrations of fibrinogen and thrombin; however, the low protein contents highly limit their use in ophthalmic surgeries due to the longer coagulation time and poor adhesion strength [17]. On the other hand, different methods have been described to prepare FGs rich in fibrinogen or thrombin [18,19,20,21,22]. These methods improve adhesion strength due to higher protein contents, but require more preparation time or specific devices.

In this study, a new method for the preparation of FGs is described. The described method enabled the preparation of FGs in less than 1 h by mixing concentrated fibrinogen and thrombin obtained from the same venipuncture blood using a simple protocol. The FGs described in this study showed enough manageability and adhesion strength to graft a conjunctival autograft on a rabbit sclera without any adverse effect. Our results indicate that 2 mL of FG could be prepared using 90 mL of donor’s blood, and could be concentrated up to 47.46 ± 9.36 mg/mL fibrinogen and 2248.12 ± 604.20 IU/mL thrombin. However, larger volumes and greater concentrations of FG can be prepared by increasing the volume of donor’s blood if needed. The developed methodology appears to offer higher fibrinogen concentrations, lower initial volumes of blood, and shorter production times than other techniques, described such as cryoprecipitation [39], other forms of chemical precipitation [40], or commercial available devices [18].

The main requirements for an FG are time-until-clot and adhesion strength [1,7]. Fibrin clots are usually formed in 10–20 min by adding calcium chloride to human plasma containing physiological concentrations of fibrinogen and thrombin. In contrast, fibrin clots are formed in seconds if isolated fibrinogen and concentrated thrombin are used [41]. The difference in clot formation times results in different adhesion strengths, because mechanical properties improve inversely proportionally to the rapidness of clot formation [36]. Mixing normal plasma and calcium chloride results in poorly adherent hydrogels with low adhesion strength (1 kPa) [7,17]. However, when calcium chloride is replaced with thrombin concentrate, as in FG1, we observed slightly higher adhesion strength (1.49 ± 0.39 kPa). Nevertheless, studies have demonstrated [17] that adhesion strength improves when fibrinogen is concentrated, as in FG2 and FG3. In our study, the adhesion strengths significantly increased up to 3.14 ± 1.09 kPa (FG3), almost 63% of the adhesion strength of the commercial FG, despite using half the fibrinogen concentration. 

In accordance with previous studies [17], FG1 showed poor results when used for autograft adhesion. In contrast, autografts glued with FG2 and FG3 showed firmer adhesion and adequate adhesion strength to maintain wound integrity as with commercial FG.

Clinical follow-up exhibited adequate ocular tolerance, with no adverse effect on the cornea and conjunctiva when FG1, FG2, or FG3 were used. In contrast, commercial FG showed inflammatory infiltrates at the graft site and within the surrounding conjunctiva. This could be attributed to a xenogeneic response to the higher human fibrinogen content of the commercial FG. The inflammation was further confirmed in the histological and immunofluorescence analysis, where commercial FG showed a mild to severe infiltration of immune cells, fibrin-clot residues, and granulation tissue. On the other hand, FG1, FG2, and FG3 samples showed normal conjunctival stroma and epithelium without the presence of immune cells, edema, scars, fibrin-clot residues, or granulation tissue.

Overall, we have developed a new method for FG manufacture that can be prepared in less than 1 h by mixing concentrated fibrinogen and thrombin obtained from a single venipuncture blood sample. However, in order to transfer the results into clinical applications, FGs should be prepared and dispensed under strict control by a hospital pharmacy service in order to guarantee patient safety. Nevertheless, due to the simplicity of our method, FGs could easily be prepared in any standard pharmacy workstation designed for non-hazardous drug preparation, allowing rapid clinical transferability. Additionally, although the method for FG preparation was initially designed for use in ophthalmology, where the demand for volume/surface ratio is low, the method could be extrapolated to other areas by increasing the amount of venipuncture blood.

## 5. Conclusions

The use of locally manufactured FGs has been shown to effectively seal a conjunctival autograft, demonstrating sufficient mechanical properties for it to be considered as a clinically useful alternative to commercial FGs for ophthalmic surgical applications. The method for FG preparation is sufficiently simple to enable on-demand FGs manufacture and dispensing by any hospital pharmacy service and can be fine-tuned for any specific need by adjusting the fibrinogen concentration and thrombin activity. 

## Data Availability

All the obtained data used to support the findings of this study are available from the corresponding author upon reasonable request.
